# Preliminary Assessment of Muscle Activity and Muscle Characteristics during Training with Powered Robotic Exoskeleton: A Repeated-Measures Study

**DOI:** 10.3390/healthcare9081003

**Published:** 2021-08-05

**Authors:** Sung-Hyeon Kim, Ho-Jin Shin, Hwi-Young Cho

**Affiliations:** 1 Department of Health Science, Gachon University Graduate School, Incheon 21936, Korea; gpgkorea30@gmail.com (S.-H.K.); gpgkorea89@gmail.com (H.-J.S.); 2 Department of Physical Therapy, Gachon University, Incheon 21936, Korea

**Keywords:** exoskeleton device, robotics, physical functional performance, locomotion, orthosis

## Abstract

A variety of robotic exoskeletons have been developed for patients with spinal cord injuries. However, the optimal training method and period for using a robotic exoskeleton have been uncertain until now. The purpose of this study is to determine the minimum training period for using a robotic exoskeleton with minimal muscle activity by investigating the changes in muscle activity and muscle characteristics of healthy adults during robotic exoskeleton training. A total of 16 people participated in the study. The robotic exoskeleton locomotion training consisted of three 50-min sessions a week for 7 weeks. The assessment consisted of sitting, standing, wide standing, sit-to-stand, and stand-to-sit where muscle activity and muscle characteristics were measured during each motion. All measurements were performed in the first session and every five sessions. Participants showed decreased muscle activity up to 10 sessions of training in the standing position, and 15 sessions in sit-to-stand and stand-to-sit motions. Upper extremity muscles showed decreased muscle activity, tone, stiffness, and logarithmic decrement up to the 15th session. The study results show that at least 15 training sessions are required to use the robotic exoskeleton with minimal load on the musculoskeletal system, and longer training is required for patients with spinal cord injury.

## 1. Introduction

The worldwide annual incidence of spinal cord injury (SCI) is 367.2–526 per million people, which signifies that there are about 2,200,000–3,150,000 new patients each year [1,2,3,4]. SCI patients experience severe limitations in daily activities, such as loss of mobility and shoulder pain related to the use of devices [5]. One of the ways to improve their quality of life is by enhancing mobility, and as one of the methods for this, the development and commercialization of the exoskeleton have been carried out [6,7,8].

In the past 10 years of research on gait training using various robotic exoskeletons for SCI patients, it has been reported that independent gait is possible with 8–20 training sessions [9,10,11,12,13,14]. As such, the robotic exoskeleton could enhance the walking ability of SCI patients, in particular, the possibility of independent activity through the robot exoskeleton in patients with complete sensorimotor SCI depends on whether it is possible to perform movements with only the upper extremity. Therefore, it is essential to establish a standardized training criterion that enables independent activities only with the upper extremity functions. However, in the studies of robotic exoskeleton so far, the methods for evaluating the subjects’ gait ability and independence are different, and also the subjects, training methods, training duration, and the number of sessions are all unclear [6,8].

The robotic exoskeleton assists or replaces movement by using the power of the device, and enables SCI patients to perform activity daily of living and gait without recruitment of lower extremity muscles. SCI patients use their upper extremities more for locomotion than healthy adults, and this leads to fatigue and damage to the musculoskeletal system in the upper extremity, shoulder, and trunk [5,15,16,17,18,19]. For SCI patients, high energy demands and fatigue limit independent activities [20,21]. Therefore, sufficient training is required for SCI patients to use the robotic exoskeleton without physical burden or damage. Since SCI patients have a different level of body function according to the type and level of injury, the training period required to use the robotic exoskeleton is different. Therefore, a standardized training protocol for the use of exoskeleton walking robots was inconsistent until the present [9,10,11,12,13,14]. In order to develop a standardized training protocol for SCI, it is preferentially necessary to establish a standard of a common minimum training period. Measurement of muscle activity or muscle characteristics can evaluate the level of proficiency in using the equipment [22]. However, in patients with SCI, since factors such as abnormal muscle tone can affect the accurate evaluation [23], assessing muscle activity or muscle characteristics in healthy adults is suitable for confirming proficiency in using the robotic exoskeleton. Therefore, identifying the training period in which the robotic exoskeleton is available with minimal effort in healthy adults can be useful in establishing a minimum training period for SCI patients.

The purpose of this study is to determine the minimum training period for using a robotic exoskeleton with minimal muscle activity by investigating the changes in muscle activity and muscle characteristics of healthy adults during robotic exoskeleton training.

## 2. Materials and Methods

### 2.1. Participants

The study population was recruited with an advertisement and poster on a bulletin board. Data collection occurred from 26 August to 20 October 2019. The inclusion criteria were as follows: (1) Anyone who has not experienced a robotic exoskeleton, (2) anyone who is sedentary or mildly active that does not engage in exercise more than two times a week, 30 min a day, and (3) anyone who has not experienced a systematic training program. The exclusion criteria were as follows: (1) Anyone who does not meet the physical requirements for a robotic exoskeleton, and (2) anyone with physical dysfunction such as musculoskeletal damage in the lower extremity. The physique condition to be fully fit to the robotic exoskeleton is shown in Appendix A. A total of 20 volunteers participated in the study. This study was approved by the Gachon University Institutional Review Board (1044396-201907-HR-127-01) and followed the Declaration of Helsinki. The study was enrolled in the Clinical Research Information Service in compliance with the World Health Organization International Clinical Trials Registry Platform (KCT0005155). All participants signed a consent form after hearing enough explanation about the study.

The sample size was calculated using G-power software (version 3.1.9.4, Heinrich Heine University, Dusseldorf, Germany) [24]. The effect size of 0.30 was obtained through data (unpublished data) of the pilot study conducted before this study, and the sample sizes were calculated by setting the alpha level to 0.05 and power to 0.8. Based on this value, 15 subjects were required, and 19 subjects were required considering a dropout rate of 20%.

### 2.2. Study Design

The study had a repeated-measures design. All study processes took place in a laboratory setting at Gachon University. All training was executed by a researcher with more than 5 years of clinical experience and a Master’s degree or above. All measurements and analyses were performed by two researchers with a Master’s degree or above who were blinded on the description and purpose of this study.

### 2.3. Exoskeleton

The robotic exoskeleton used in the study is the Robowear10 (NT Robot, Co. Seoul, Korea), and the detailed structure of the equipment is shown in Appendix A. The joints of the Robowear10 can produce 125 Nm of rated torque and 271 Nm of peak torque. It is designed for use by up to 100 kg of users. Its maximum walking speed is 1.0 m/s, and it can be used for up to 60 min during continuous walking. Robowear10 fastens body segments with straps and buckles. Robowear10 is operated with an operation button in a wrist band and is also designed to enable movement control in the hip joint and knee joint manually with an operation button outside the device.

The operation unit consists of the following five buttons: (1) Connection and disconnection with the device, (2) battery level check, (3) sit to stand, (4) walking, and (5) stand to sit. To maintain balance, assistance using parallel bars, a walker or crutches is required for all movements.

### 2.4. Training Procedure

Before participating in training, anthropometric measurements of the participants were obtained. The locomotor training program with the robotic exoskeleton consisted of a total of 20 sessions of 50 min per session, three times a week [9,14]. During this period, participants were trained under the management of certified therapists, and the training goals and detailed training information for each session are shown in Appendix A. In all training sessions, participants were instructed to perform with the upper extremities only, without using the lower extremities as much as possible.

### 2.5. Outcome Measure

Measurements of muscle activity and muscle characteristics were performed in the first session and every 5 sessions. Muscle activity was obtained under the following conditions: (1) sitting posture (SIT), (2) standing posture (STD), (3) wide standing posture (WSTD), (4) sit to stand (StSTD), and (5) stand to sit (STDtS). Muscle characteristics were assessed under the following conditions: (1) SIT, (2) STD, and (3) WSTD. SIT is a resting posture in which the subject sits without moving, and STD is a state in which the subject maintains a standing posture using the crutch. The distance between the crutch and the toe is 25 cm. WSTD is a position where the subject stands wide using the crutch, the distance between the crutch and the toe is 50 cm. StSTD is a motion in which the subject rises from a sitting position to a standing position using an exoskeleton robot, whereas STDtS is a motion in which the subject sits in a chair from a standing position. All subjects took the SIT measurement first and then performed the suggested movements according to the random order of the researcher.

All subjects received prior guidance and experience on the measurement method before participation in this study in order to confirm the familiarization of the muscle activity and muscle characteristics measurement method and the adverse effects on electrode attachment. All assessments were conducted in a separate bright room (temperature: 22–24 °C) independent from the outside in the morning of the next day after each standard training session to prevent fatigue based on training. In addition, subjects were given a 1 min rest time between each trial and a 5 min rest time between each condition during the assessment. The assessment of muscle activity and muscle characteristics in each posture was repeated three times, and the average was obtained.

#### 2.5.1. Muscle Activity

Muscle activity was measured using 8-channel wireless electromyography (Noraxon Telemyo 2400 T, NORAXON Inc., Scottsdale, AZ, USA) with high intra-rater reliability (0.81–0.98) [25]. The skin sites were shaved, abraded and cleaned with isopropyl alcohol to reduce source impedance. Electrodes were attached to the muscles on the predominant side of the participant. For the raw electromyogram signal, band pass filter was set to 20–350 Hz, and after applying rectification, root mean square (RMS) window size was set to 100 ms. The RMS value was normalized to reference voluntary contraction. Muscle activity was measured in all five postures. Data of SIT, STD, and WSTD were measured while the subject maintained each posture for 1 min, and data of StSTD and STDtS were measured while performing each movement using a robotic exoskeleton. All data were calculated as mean values of muscle activity during each motion. Electrodes were attached to the following locations [26,27,28,29,30]: (1) Rectus femoris (RF), (2) tibialis anterior (TA), (3) biceps femoris (BF), (4) gastrocnemius (GCM), (5) deltoid (DEL), (6) upper trapezius (UT), (7) middle trapezius (MT), and (8) erector spine lumbar (ESL).

#### 2.5.2. Muscle Characteristics

Muscle characteristics were measured using a hand-held myotonometer (Myoton AS, Tallinn, Estonia), which shows high reliability (0.86–0.94) [31,32,33]. Muscle characteristics were measured from seven muscles where the muscle activity was measured from, excluding the waist, which is covered by the equipment. The location of measurement was identical to where music activity was measured. Measured muscle characteristics are as follows: (1) tone (tone, Hz), (2) stiffness (N/m), (3) elasticity (logarithmic decrement, Log-D). Muscle tone describes the state of tension of a muscle. Muscle stiffness is the biomechanical property of a muscle that characterizes the resistance of a contraction to an external force that deforms its initial shape. Muscle elasticity is the biomechanical property of a muscle that characterizes the ability to recover its initial shape after a contraction or removal of an external force of deformation. To reduce measurement errors, the measuring part was marked with a marker prior to the first measurement, and measurements were taken again at the marked spot. The equipment was positioned perpendicular to the surface of the skin when measurements were taken. All repeated measurements were performed by the same researcher.

### 2.6. Statistical Analysis

All variables used the mean value of data from three repeated measurements, and the results were presented as the mean ± standard deviation. Statistical analysis was performed using the SPSS 25.0 (SPSS Inc., Chicago, IL, USA). Normal distribution of the data was assessed with the Shapiro–Wilk test. Repeated measures (within-participant analysis; 5 times) analysis of variance (ANOVA) followed by Bonferroni’s post hoc multiple comparisons test was used to compare variables following normality. Sphericity was confirmed by Mauchly’s sphericity test. In the case of spherical violations, the Greenhouse–Geisser estimate of degrees of freedom was used. Statistical significance was accepted at the level of 0.05.

## 3. Results

### 3.1. General Characteristic of Participants

**Figure 1:**
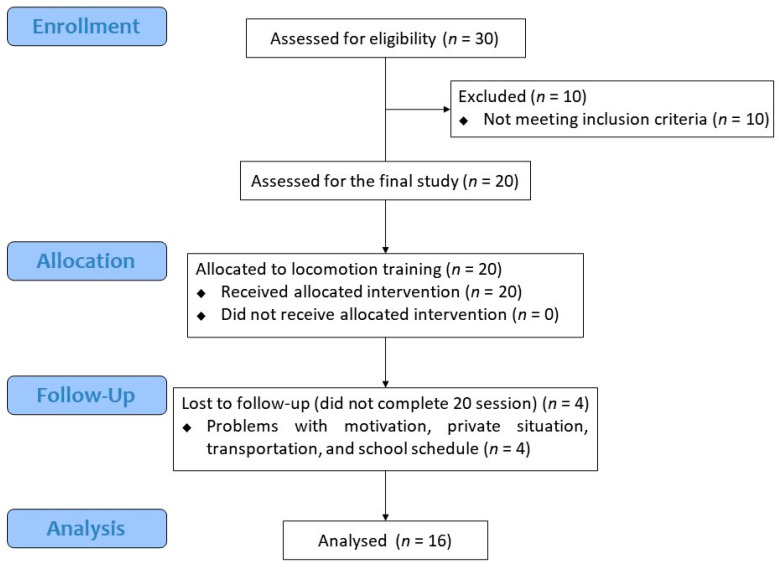
Consort flow diagram.

The overall research process is shown in Figure 1. There were 20 participants who met the eligibility criteria. Among them, 16 participants (80%) completed the training protocol, and four participants (20%) were dropped out. The reason for drop-out is that they did not complete all training sessions (less than 5 sessions: 2, less than 10 sessions: 2). Finally, data on the 16 participants were used in the analysis (Table 1). There were no adverse events during the entire training period.

### 3.2. Muscle Activity

#### 3.2.1. Static Condition

**Figure 2:**
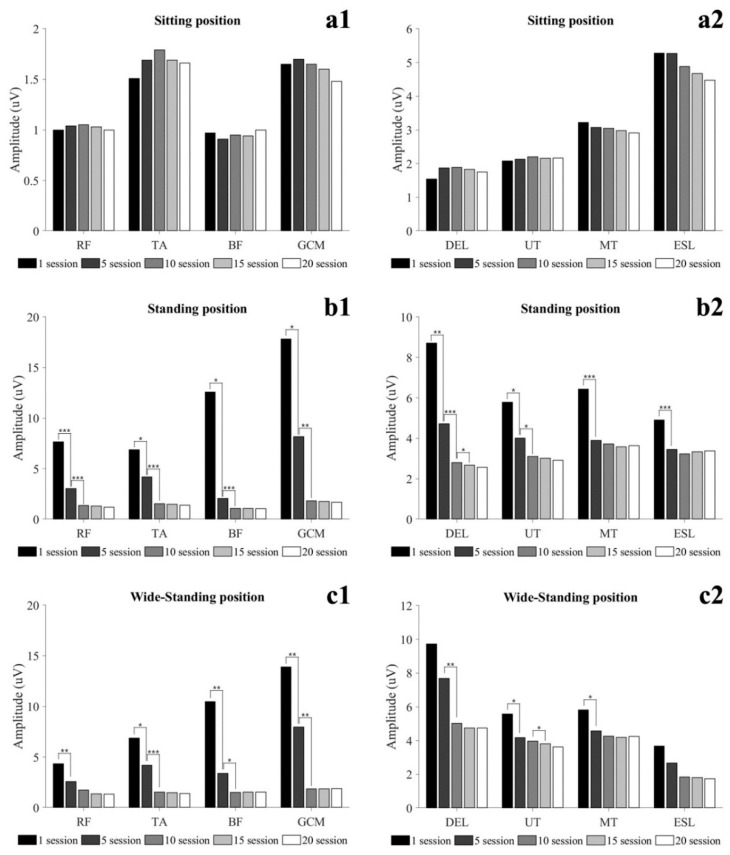
Changes in muscle activity in static condition during locomotion training. (**a1**). Sitting (lower extremity muscles). (**a2**). Sitting (trunk and shoulder muscles). (**b1**). standing (lower extremity muscles). (**b2**). standing (trunk and shoulder muscles). (**c1**). wide-standing (lower extremity muscles). (**c2**). wide-standing (trunk and shoulder muscles); * = *p* < 0.05, ** = *p* < 0.01, *** = *p* < 0.001. Abbreviation: RF, rectus femoris; TA, tibialis anterior; BF, biceps femoris; GCM, gastrocnemius; DEL, deltoid; UT, upper trapezius; MT, middle trapezius; ESL, erector spine lumbar.

Muscle activity in static conditions is shown in Figure 2 and Table A1. There was no significant difference between training sessions in all muscle activity in SIT (*p* > 0.05). In the STD, all muscles activity was significantly decreased at 5 sessions, and lower leg muscles, DEL and UT were significantly decreased at 10 sessions (*p* < 0.05). In 15 sessions, only DEL was significantly reduced (*p* < 0.05). In WSTD, lower extremity muscle, UT and MT were significantly decreased at 5 sessions, and TA, GCM, and DEL were significantly decreased at 10 sessions (*p* < 0.05). Only UT significantly decreased at 15 sessions (*p* < 0.05).

#### 3.2.2. Dynamic Condition

**Figure 3:**
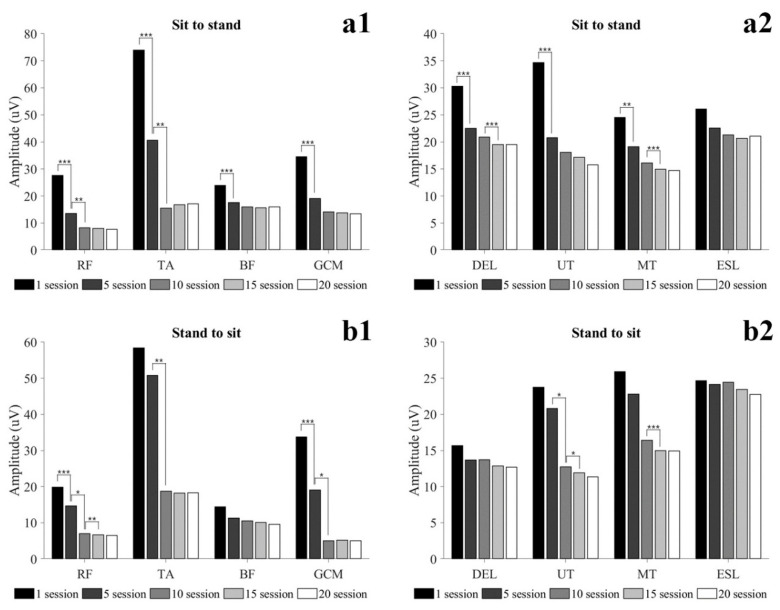
Changes in muscle activity in dynamic condition during locomotion training. (**a1**). sit to stand (lower extremity muscles). (**a2**). sit to stand (trunk and shoulder muscles). (**b1**). stand to sit (lower extremity muscles). (**b2**). stand to sit (trunk and shoulder muscles); * = *p* < 0.05, ** = *p* < 0.01, *** = *p* < 0.001. Abbreviation: RF, rectus femoris; TA, tibialis anterior; BF, biceps femoris; GCM, gastrocnemius; DEL, deltoid; UT, upper trapezius; MT, middle trapezius; ESL, erector spine lumbar.

Muscle activity in dynamic conditions is shown in Figure 3 and Table A2. In StSTD, all muscles activity except ESL was significantly decreased at 5 sessions, and RF and TA were significantly decreased at 10 sessions (*p* < 0.05). At 15 sessions, DEL and MT were significantly decreased (*p* < 0.05). In STDtS, RF and GCM were significantly decreased at 5 sessions, and RF, TA, GCM and UT were significantly decreased at 10 sessions (*p* < 0.05). In addition, RF, UT and MT were significantly decreased at 15 sessions (*p* < 0.05).

### 3.3. Muscle Characteristic

**Figure 4:**
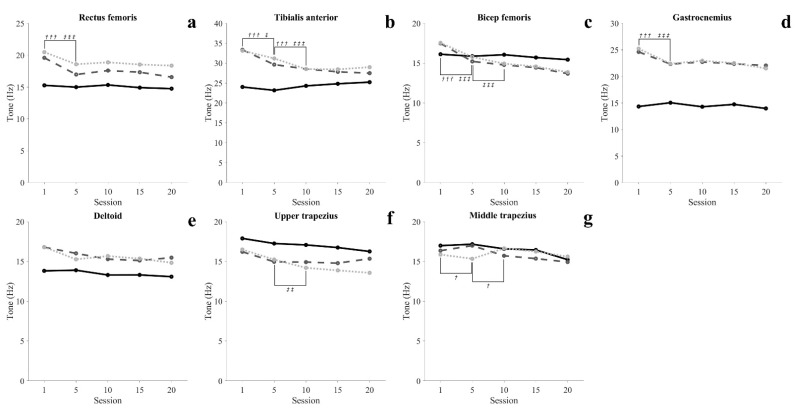
Time-series graphs of significant difference periods for muscle tone (sitting position: solid line; standing position: dashed line; wide standing position: dotted line). ^†^ Significant differences in a standing position, ^†^ = *p* < 0.05, ^†††^ = *p* < 0.001. ^‡^ Significant differences in a wide standing position, ^‡^ = *p* < 0.05, ^‡‡^ = *p* < 0.01, ^‡‡‡^ = *p* < 0.001.

**Figure 5:**
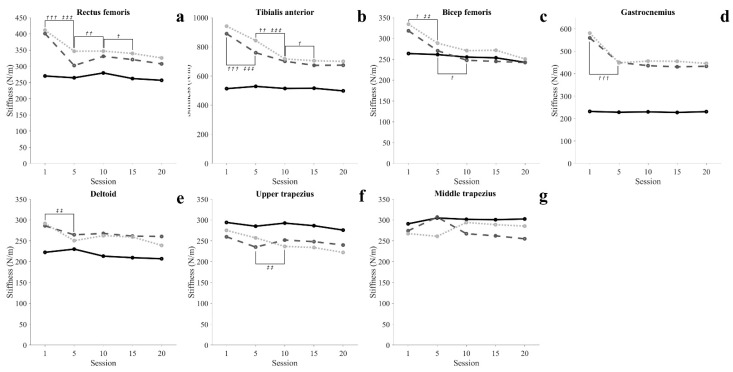
Time-series graphs of significant difference periods for muscle stiffness (sitting position: solid line; standing position: dashed line; wide standing position: dotted line). ^†^ Significant differences in a standing position, ^†^ = *p* < 0.05, ^††^ = *p* < 0.01, ^†††^ = *p* < 0.001. ^‡^ Significant differences in a wide standing position, ^‡‡^ = *p* < 0.01, ^‡‡‡^ = *p* < 0.001.

**Figure 6:**
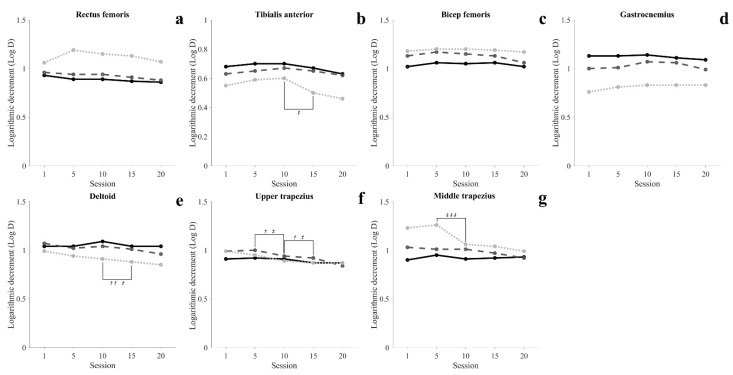
Time-series graphs of significant difference periods for muscle elasticity (sitting position: solid line; standing position: dashed line; wide standing position: dotted line). ^†^ Significant differences in a standing position, ^†^ = *p* < 0.05, ^††^ = *p* < 0.01. ^‡^ Significant differences in a wide standing position, ^‡^ = *p* < 0.05, ^‡‡‡^ = *p* < 0.001.

Muscle characteristics are shown in Figure 4, Figure 5 and Figure 6 and Table A3, Table A4 and Table A5.

#### 3.3.1. Muscle Tone

In the SIT, there was no significant change in all muscles (*p* > 0.05). In the STD, lower extremity muscles and MT was significantly decreased at 5 sessions, and TA and MT were significantly decreased at 10 sessions (*p* < 0.05). In WSTD, lower extremity muscle was significantly decreased at 5 sessions, and TA, BF and UT were significantly decreased at 10 sessions (*p* < 0.05).

#### 3.3.2. Muscle Stiffness

In the SIT, there was no significant change in all muscles (*p* > 0.05). In the STD, lower extremity muscle was significantly decreased at 5 sessions, and RF, TA and BF were significantly decreased at 10 sessions (*p* < 0.05). At 15 sessions, RF and TA were significantly reduced (*p* < 0.05). In the WSTD, lower extremity muscle and DEL was significantly decreased at 5 sessions, and TA and UT were significantly decreased at 10 sessions (*p* < 0.05).

#### 3.3.3. Muscle Elasticity

In the SIT, there was no significant change in all muscles (*p* > 0.05). In the STD, UT was significantly decreased at 10 sessions, and DEL and UT were significantly decreased at 15 sessions (*p* < 0.05). In the WSTD, UT and MT were significantly decreased at 10 sessions, and DEL and UT were significantly decreased at 15 sessions (*p* < 0.05).

## 4. Discussion

This study was conducted to determine the minimum training period for using the robotic exoskeleton with minimal muscle activity by investigating the changes in muscle activation and muscle characteristics in healthy adults during robotic exoskeleton training.

SCI patients with paraplegia have to use aids such as hip–knee–ankle–foot orthosis to perform gait and functional movements. In order to perform these functional movements using an assistive device, the subject mainly relies on upper extremity and shoulder movement. Therefore, the possibility of independent activity through the robotic exoskeleton in SCI patients depends on the functional level and the ability of the upper extremities to perform functional movements using assistive devices. This study conducted a 7-week training protocol consisting of 50 min per session, 3 times a week for healthy adults. In the results of this study, muscle activity in STD tended to decrease with the progress of training. In addition, after 10 sessions, STD was maintained with muscle activity similar to SIT of TA, BF, and GCM. RF showed muscle activity similar to SIT after 15 sessions in WSTD, and after 20 sessions in STD. Additionally, the muscle tone of all lower extremity muscles decreased as training progressed, and was maintained steadily after 10 sessions (Figure 4(a1–a4)). In particular, BF showed a similar tone in the STD and SIT after 5 sessions. In the StSTD and STDtS, the muscle activity of RF continuously decreased until 15 sessions and maintained a steady (Figure 3(b1)). Such results imply that in order to maintain static activities (SIT, STD, WSTD) without mobilizing the lower extremity, at least 10 sessions of training are required, and dynamic activities (StSTD, STDtS) require at least 15 training sessions. The results of the study show that healthy adults gradually reduced the assistance by the lower extremities with the improvement of proficiency during exoskeleton robot training. However, SCI patients with motor and sensory deficits are not provided with assistance by lower extremities like healthy people [34]. In addition, in patients with SCI, the fear of falling may lead to a self-imposed decline in activity and function [35]. Because of these effects, patients with SCI may require a longer duration of training than results seen in healthy adults.

The activity of muscles in the upper extremity and trunk all showed a decreasing tendency in all conditions as training progressed, and maintained a steady level after 15 sessions. In particular, in 20 sessions, STD was maintained with muscle activity similar to SIT of DEL. These changes in muscle activity in the upper extremity and trunk show that at least 15 training sessions are required to achieve proficiency to perform movements with the minimum energy demand. Elasticity (Log-D) indicates the degree of loss of mechanical energy, and decreased Log-D signifies conservation of mechanical energy and increases in elasticity [36,37]. In our results, the Log-D values of upper extremity muscles decreased as training progressed. The MT maintained a steady level after 10 sessions, and the DEL and UT maintained a steady level after 15 sessions (Figure 6(c5–c7)). Such results imply that the amount of energy loss during movement decreases as proficiency increases, and support the argument that at least 15 training sessions are required to perform movements with efficient energy demand. Unlike other lower extremity muscles, GCM showed a high tone in standing movements throughout the entire training period compared to SIT. This is seen as a reflex caused by muscles being stretched beyond their normal length in STD in which the body leans forward [38,39,40]. Like this, involuntary changes in the length of muscles may cause changes in the mechanical characteristics of muscles [41].

The musculoskeletal pain that occurs while using a wheelchair or crutch for locomotion is one of the most common problems in SCI patients. Jain et al. [5] revealed that SCI patients using a crutch or cane for locomotion complain of shoulder pain the most. One of the major causes that trigger such pain is the overuse of the upper extremities for locomotion [15,16,17,18]. In order to perform gait and functional movements using the robotic exoskeleton, the user is given weight and the weight of the robot to the upper extremity, and hyperactivity of the muscles in the relevant area is required until the user becomes proficient in using the robot. This could often lead to musculoskeletal problems in the upper extremities and shoulders. In addition, higher stiffness of the muscle limits mobility and may result in increased injury risk [42,43,44]. Our results showed that the muscle activity and muscle stiffness of the upper extremity in healthy adults decreased as the training progressed 3 times per week and maintained constant after 15 sessions (Figure 5). Sylos-Labini et al. [19] reported that SCI patients use upper extremity muscles more than healthy people when using an exoskeleton as well. Based on this evidence, it could be inferred that a longer period of training is required than the results of this study in order to obtain proficiency in manipulating the robotic exoskeleton with minimal upper extremity muscle activity in SCI patients.

To date, various studies have reported training using the robotic exoskeleton, but clear training standards and methods are not yet clear. While some studies have reported that at least 20 sessions or more training is required for subjects to independently use the robotic exoskeleton [9,14], some studies have suggested that independent walking using the robotic exoskeleton is possible even with 6–9 sessions of training [10,13]. Bass et al. [12] suggested that it is required to identify a training period and method that could minimize the load on the upper extremities and shoulders in order to properly perform gait and functional movements using the robotic exoskeleton and to prevent musculoskeletal damage. In this study, subjects were instructed to perform functional training 3 times a week, and after 15 training sessions for 5 weeks in healthy adults, the muscle activity and muscle characteristics of the upper and lower extremities for performing functional movements were confirmed to be minimized. According to our results, it could be inferred that at least 15 sessions or more training sessions are needed to minimize the burden on the musculoskeletal system when patients with SCI and other neurological or musculoskeletal injuries perform functional movement training using the robotic exoskeleton.

### Limitations

There are a few limitations to this study. First, this study conducted interventions and evaluations on healthy adults, not SCI patients, so there is a limit to applying the results of this study to patients with SCI or other neurological and musculoskeletal problems. However, since the results are drawn from healthy adults, it may be appropriate as a guideline to design systematic training plans and periods for less capable patients. Second, a small number of samples were used for analysis. Because of the small number of samples used, care should be taken in generalizing the study results. Third, it did not compare training with other robotic exoskeletons. As there are differences in terms of the weight, size, and motor output of robotic exoskeletons, it is difficult to generalize. Additional studies on locomotion training of SCI patients using the robotic exoskeleton and comparative studies with different robotic exoskeletons are needed Fourth, we trained healthy adults to conduct 3 times a week for 7 weeks and suggested that a minimum of 15 training sessions is required as the minimum training period. However, we did not perform a randomized trial for comparison with other gait aids and training protocols. For this reason, the results may vary depending on the difference between our method and other exercise types or modalities, frequency, intensity, time (duration), volume (dosage). In addition, in order to induce hypertrophy in patients with SCI, a longer period of training is required than the results of this study obtained for healthy adults. Finally, measurement of normalized muscle activity through a maximum voluntary isometric contraction was not performed. Therefore, it was not possible to compare this study with a study using other equipment and patients. In addition, we did not evaluate previous muscle mass and strength in the subjects, and this may play a relevant factor in the minimum time period and dosage required for the intended adaptations in the training of the robotic exoskeleton.

## 5. Conclusions

We trained healthy adults to conduct functional movements using the robotic exoskeleton 3 times a week for 50 min for 7 weeks and found that at least 15 training sessions were required for the subject to use the robot with minimal muscle activity. Therefore, we suggest that patients with SCI or other nervous system damage with lower physical functions compared to healthy people need at least 15 training sessions to minimize the burden on the musculoskeletal system when performing gait and functional movements using the robotic exoskeleton.

## Figures and Tables

**Table 1:** General characteristics of participants.

Parameters		Value (N = 16)
Gender, male/female (n) ∗		7/9
Age (year) ^†^		24.88 ± 2.03
Body height (cm) ^†^		166.06 ± 8.00
Body mass (kg) ^†^		59.63 ± 8.71
Body mass index (kg/m^2^) ^†^		21.51 ± 1.69
Total leg length (cm) ^†^	Rt.	85.69 ± 5.48
	Lt.	85.59 ± 5.67
Thigh length (cm) ^†^	Rt.	37.21 ± 2.48
	Lt.	37.16 ± 2.54
Tibia length (cm) ^†^	Rt.	37.21 ± 2.60
	Lt.	37.15 ± 2.43

Rt., Right side; Lt., Left side; ∗ Values are expressed as number (n); ^†^ Values are expressed as mean + SD.

## Data Availability

The data presented in this study are available on request from the corresponding author.

## Appendix A

**Table A1:** Muscle activity in static condition during the locomotion training protocol.

Variables	1 Session	5 Session	10 Session	15 Session	20 Session	η^2^	*p*-Value
**Sitting (uV)**							
Rectus femoris	1.00 (0.22)	1.04 (0.25)	1.05 (0.27)	1.03 (0.28)	1.00 (0.34)	0.049	0.493
Tibialis anterior	1.51 (0.72)	1.69 (0.88)	1.79 (0.98)	1.69 (0.87)	1.66 (0.99)	0.127	0.133
Biceps femoris	0.97 (0.30)	0.91 (0.25)	0.95 (0.29)	0.94 (0.29)	1.00 (0.43)	0.072	0.324
Gastrocnemius	1.65 (0.54)	1.70 (0.51)	1.65 (0.44)	1.60 (0.45)	1.48 (0.45)	0.133	0.117
Deltoid	1.54 (0.36)	1.87 (0.75)	1.89 (0.51)	1.83 (0.54)	1.75 (0.56)	0.147	0.099
Upper trapezius	2.08 (0.26)	2.13 (0.56)	2.20 (0.31)	2.16 (0.49)	2.17 (0.58)	0.020	0.822
Middle trapezius	3.22 (0.97)	3.07 (0.82)	3.05 (1.10)	2.98 (0.85)	2.91 (1.20)	0.079	0.289
Erector spine	5.28 (3.71)	5.27 (3.98)	4.88 (4.47)	4.67 (4.02)	4.47 (4.17)	0.037	0.501
**Standing (uV)**							
Rectus femoris	7.66 (4.82)	3.04 (1.39) ***	1.37 (0.42) *^†††^*	1.32 (0.40)	1.20 (0.33)	0.629	<0.001
Tibialis anterior	6.88 (5.62)	4.19 (1.79) *	1.54 (0.61) *^†††^*	1.49 (0.58)	1.41 (0.64)	0.502	0.001
Biceps femoris	12.58 (17.14)	2.05 (0.87) *	1.07 (0.20) *^†††^*	1.05 (0.17)	1.06 (0.30)	0.320	0.018
Gastrocnemius	17.82 (18.54)	8.17 (8.05) *	1.82 (0.60) *^††^*	1.76 (0.54)	1.70 (0.62)	0.422	0.003
Deltoid	8.71 (4.01)	4.72 (1.46) **	2.80 (1.14) *^†††^*	2.67 (1.02) *^‡^*	2.41 (1.06)	0.715	<0.001
Upper trapezius	5.78 (1.89)	4.02 (1.56) *	3.10 (0.80) *^†^*	3.02 (0.98)	2.92 (1.23)	0.592	<0.001
Middle trapezius	6.43 (2.55)	3.90 (0.40) ***	3.72 (0.39)	3.58 (0.44)	3.64 (0.73)	0.569	<0.001
Erector spine	4.90 (2.68)	3.45 (2.09) ***	3.24 (1.95)	3.34 (2.24)	3.38 (2.67)	0.439	<0.001
**Wide-standing (uV)**							
Rectus femoris	4.33 (4.10)	2.59 (2.69) **	1.74 (1.13)	1.37 (0.74)	1.34 (0.70)	0.317	0.014
Tibialis anterior	10.48 (8.80)	3.38 (2.53) *	1.50 (0.56) *^†††^*	1.53 (0.65)	1.54 (0.75)	0.504	0.001
Biceps femoris	11.27 (16.77)	1.76 (0.87) **	1.34 (0.64) *^†^*	1.30 (0.60)	1.20 (0.53)	0.273	0.031
Gastrocnemius	13.91 (11.06)	7.98 (6.32) **	1.87 (0.79) *^††^*	1.87 (0.94)	1.90 (1.08)	0.537	<0.001
Deltoid	9.73 (4.57)	7.69 (2.53)	5.02 (1.87) *^††^*	4.74 (1.79)	4.74 (2.42)	0.394	0.002
Upper trapezius	5.57 (1.88)	4.18 (1.33) *	3.96 (0.92)	3.82 (1.00) *^‡^*	3.63 (1.21)	0.392	0.003
Middle trapezius	5.81 (2.16)	4.58 (1.35) *	4.26 (0.77)	4.20 (0.81)	4.24 (1.49)	0.313	0.007
Erector spine	3.68 (3.38)	2.68 (1.76)	1.85 (0.38)	1.80 (0.36)	1.74 (0.56)	0.218	0.041

Data presented as mean (standard deviation); η^2^, partial eta square; * *p* < 0.05, ** *p* < 0.01, *** *p* < 0.001, vs. 1-session; *^†^**p* < 0.05, *^††^* *p* < 0.01, *^†††^* *p* < 0.001, vs. 5-session; *^‡^ p* < 0.05, vs. 10-session.

**Table A2:** Muscle activity in dynamic condition during the locomotion training protocol.

Variables	1 Session	5 Session	10 Session	15 Session	20 Session	η^2^	*p*-Value
**Sit to stand (uV)**							
Rectus femoris	27.65 (11.55)	13.57 (7.46) ***	8.29 (3.66) *^††^*	8.05 (3.80)	7.65 (3.91)	0.738	<0.001
Tibialis anterior	73.96 (47.36)	40.66 (34.04) ***	15.56 (8.96) *^††^*	16.80 (10.97)	17.08 (12.51)	0.633	<0.001
Biceps femoris	23.92 (15.16)	17.62 (10.71) ***	15.91 (8.69)	15.66 (8.92)	15.93 (10.89)	0.457	<0.001
Gastrocnemius	34.58 (24.33)	19.12 (13.06) ***	14.08 (5.61)	13.73 (5.60)	13.48 (6.29)	0.459	0.002
Deltoid	30.28 (11.00)	22.48 (6.75) ***	20.88 (3.52)	19.50 (3.21) *^‡‡‡^*	19.49 (7.07)	0.526	<0.001
Upper trapezius	34.65 (11.22)	20.77 (7.40) ***	18.07 (4.78)	17.17 (5.63)	15.75 (5.30)	0.617	<0.001
Middle trapezius	24.55 (12.49)	19.10 (9.69) **	16.12 (4.45)	14.97 (4.25) *^‡‡‡^*	14.74 (4.42)	0.413	0.001
Erector spine	26.06 (14.19)	22.58 (9.16)	21.30 (6.64)	20.65 (6.49)	21.08 (8.62)	0.207	0.040
**Stand to sit (uV)**							
Rectus femoris	19.86 (13.26)	14.66 (12.06) ***	7.01 (3.41) *^†^*	6.69 (3.39) *^‡‡^*	6.51 (3.44)	0.515	<0.001
Tibialis anterior	58.38 (48.35)	50.81 (53.80)	18.73 (19.75) *^††^*	18.17 (19.28)	18.27 (21.02)	0.559	<0.001
Biceps femoris	14.41 (4.13)	11.28 (3.37)	10.49 (5.01)	10.11 (4.60)	9.57 (4.81)	0.191	0.051
Gastrocnemius	33.79 (21.95)	19.05 (22.08) ***	5.09 (1.77) *^†^*	5.23 (1.93)	5.08 (2.11)	0.544	<0.001
Deltoid	15.66 (6.55)	13.68 (9.32)	13.71 (5.34)	12.87 (5.04)	12.70 (6.58)	0.099	0.210
Upper trapezius	23.76 (19.13)	20.82 (11.90)	12.71 (6.00) *^†^*	11.91 (5.32) *^‡^*	11.36 (5.16)	0.290	0.015
Middle trapezius	25.92 (22.19)	22.81 (16.93)	16.40 (9.57)	14.99 (8.73) *^‡‡‡^*	14.95 (10.25)	0.228	0.038
Erector spine	24.67 (16.14)	24.16 (15.29)	24.43 (14.14)	23.44 (14.26)	22.78 (15.70)	0.020	0.733

Data presented as mean (standard deviation); η^2^, partial eta square; ** *p* < 0.01, *** *p* < 0.001, vs. 1-session; *^†^* *p* < 0.05, *^††^* *p* < 0.01, vs. 5-session; *^‡^* *p* < 0.05, *^‡‡^* *p* < 0.01, *^‡‡‡^* *p* < 0.001, vs. 10-session.

**Table A3:** Muscle tone during the locomotion training protocol.

Variables	1 Session	5 Session	10 Session	15 Session	20 Session	η^2^	*p*-Value
**Sitting (Hz)**							
Rectus femoris	15.27 (0.63)	14.99 (0.50)	15.33 (0.73)	14.91 (0.95)	14.75 (3.89)	0.021	0.609
Tibialis anterior	24.02 (3.27)	23.18 (3.08)	24.30 (3.32)	24.83 (3.53)	25.23 (4.37)	0.130	0.125
Biceps femoris	16.12 (2.41)	15.87 (1.76)	16.06 (2.12)	15.71 (1.97)	15.44 (3.24)	0.039	0.530
Gastrocnemius	14.34 (1.66)	15.05 (1.80)	14.29 (0.96)	14.74 (1.51)	13.97 (2.90)	0.062	0.373
Deltoid	13.82 (0.83)	13.90 (1.23)	13.30 (1.03)	13.31 (1.38)	13.09 (3.17)	0.069	0.322
Upper trapezius	17.90 (0.78)	17.26 (0.59)	17.08 (1.03)	16.75 (1.19)	16.26 (2.86)	0.166	0.079
Middle trapezius	16.99 (0.72)	17.17 (1.03)	16.59 (1.32)	16.45 (1.30)	15.23 (2.77)	0.200	0.051
**Standing (Hz)**							
Rectus femoris	19.59 (2.19)	16.97 (1.21) ***	17.59 (1.98)	17.33 (1.66)	16.56 (2.87)	0.311	0.005
Tibialis anterior	33.35 (2.41)	29.64 (3.37) ***	28.54 (2.96) *^†††^*	27.83 (3.11)	27.49 (5.50)	0.467	<0.001
Biceps femoris	17.48 (1.90)	15.22 (1.22) ***	14.78 (0.56)	14.44 (0.94)	13.68 (3.35)	0.405	0.001
Gastrocnemius	24.62 (1.60)	22.33 (0.62) ***	22.73 (1.40)	22.37 (1.95)	22.06 (3.91)	0.223	0.030
Deltoid	16.80 (1.79)	16.02 (2.31)	15.29 (2.37)	15.12 (2.21)	15.48 (4.75)	0.129	0.135
Upper trapezius	16.22 (1.45)	14.96 (0.69)	14.92 (0.92)	14.79 (1.09)	15.35 (2.83)	0.151	0.084
Middle trapezius	16.36 (1.32)	16.99 (1.87) *	15.72 (1.27) *^†^*	15.36 (1.46)	14.93 (3.23)	0.197	0.040
**Wide-standing (Hz)**							
Rectus femoris	20.48 (2.11)	18.58 (1.89) ***	18.88 (2.26)	18.54 (2.11)	18.36 (3.51)	0.222	0.029
Tibialis anterior	33.13 (4.15)	31.19 (2.88) *	28.52 (1.87) *^†††^*	28.45 (2.00)	28.99 (5.60)	0.351	0.002
Biceps femoris	17.55 (1.44)	15.78 (1.10) ***	14.94 (0.67) *^†††^*	14.58 (0.96)	13.87 (3.13)	0.446	<0.001
Gastrocnemius	25.23 (2.40)	22.40 (1.11) ***	22.96 (1.20)	22.51 (1.60)	21.52 (4.32)	0.299	0.006
Deltoid	16.82 (2.37)	15.27 (1.48)	15.68 (1.75)	15.36 (1.63)	14.84 (2.72)	0.162	0.056
Upper trapezius	16.50 (2.39)	15.24 (0.96)	14.20 (0.84) *^††^*	13.88 (1.02)	13.57 (2.87)	0.341	0.003
Middle trapezius	15.87 (0.95)	15.34 (0.93)	16.65 (0.92)	16.25 (0.90)	15.61 (2.93)	0.116	0.168

Data presented as mean (standard deviation); η^2^, partial eta square; * *p* < 0.05, *** *p* < 0.001, vs. 1-session; *^†^* *p* < 0.05, *^††^* *p* < 0.01, *^†††^* *p* < 0.001, vs. 5-session.

**Table A4:** Muscle stiffness during the locomotion training protocol.

Variables	1 Session	5 Session	10 Session	15 Session	20 Session	η^2^	*p*-Value
**Sitting (N/m)**							
Rectus femoris	270.01 (12.25)	264.49 (12.37)	279.41 (14.23)	262.11 (18.76)	256.81 (53.39)	0.114	0.174
Tibialis anterior	513.28 (91.50)	528.81 (89.06)	514.48 (87.40)	516.15 (99.95)	497.98 (141.07)	0.042	0.463
Biceps femoris	263.97 (32.51)	261.38 (34.43)	255.33 (33.34)	253.67 (34.32)	242.71 (63.49)	0.086	0.261
Gastrocnemius	232.08 (22.11)	228.41 (17.10)	230.18 (20.99)	227.56 (24.36)	231.09 (55.77)	0.006	0.840
Deltoid	222.64 (17.36)	230.27 (18.24)	213.37 (26.87)	209.76 (17.10)	207.16 (38.09)	0.166	0.063
Upper trapezius	294.40 (15.16)	285.33 (15.88)	292.81 (22.76)	286.56 (24.69)	276.04 (54.07)	0.080	0.281
Middle trapezius	291.12 (28.77)	305.19 (16.63)	301.87 (30.47)	301.00 (43.53)	302.71 (50.82)	0.043	0.556
**Standing (N/m)**							
Rectus femoris	400.99 (71.61)	302.31 (24.27) ***	330.65 (39.88) *^††^*	320.65 (39.00) *^‡^*	307.43 (68.13)	0.525	<0.001
Tibialis anterior	889.74 (108.39)	759.43 (136.05) ***	700.93 (129.42) *^††^*	673.03 (142.88) *^‡^*	673.92 (211.43)	0.538	<0.001
Biceps femoris	318.46 (80.94)	270.64 (38.11) *	247.79 (30.19) *^†^*	245.01 (33.12)	242.34 (50.43)	0.369	0.001
Gastrocnemius	559.65 (81.38)	450.33 (41.33) ***	436.00 (56.68)	430.88 (61.12)	433.41 (85.84)	0.461	<0.001
Deltoid	286.26 (52.00)	264.71 (46.63)	267.97 (65.48)	261.55 (59.89)	260.66 (81.61)	0.094	0.227
Upper trapezius	259.90 (31.01)	235.24 (33.13)	252.12 (26.44)	248.20 (32.06)	240.12 (54.55)	0.072	0.331
Middle trapezius	274.33 (21.15)	307.30 (53.72)	267.29 (30.94)	262.24 (34.01)	255.10 (75.96)	0.191	0.058
**Wide-standing (N/m)**							
Rectus femoris	411.48 (67.10)	346.49 (43.44) ***	346.84 (38.64)	339.63 (36.98)	325.73 (54.49)	0.463	<0.001
Tibialis anterior	941.58 (184.07)	842.82 (138.11) ***	717.02 (121.66) *^†††^*	704.91 (132.77)	700.11 (209.44)	0.549	<0.001
Biceps femoris	334.29 (56.76)	288.70 (41.35) **	270.92 (37.42)	271.78 (36.27)	250.80 (51.97)	0.443	<0.001
Gastrocnemius	580.98 (104.10)	448.66 (50.98)	456.38 (37.59)	455.36 (42.77)	446.14 (103.84)	0.486	<0.001
Deltoid	291.97 (54.510)	250.63 (37.72) **	262.56 (41.83)	259.54 (39.65)	239.23 (48.97)	0.279	0.005
Upper trapezius	275.36 (51.78)	257.31 (18.52)	236.81 (10.98) *^††^*	234.18 (18.00)	222.20 (52.03)	0.310	0.003
Middle trapezius	267.57 (25.93)	261.10 (29.66)	294.31 (18.53)	289.07 (16.96)	285.64 (65.12)	0.142	0.118

Data presented as mean (standard deviation); η^2^, partial eta square; * *p* < 0.05, ** *p* < 0.01, *** *p* < 0.001, vs. 1-session; *^†^ p* < 0.05, *^††^* *p* < 0.01, *^†††^* *p* < 0.001, vs. 5-session; *^‡^ p* < 0.05 vs. 10-session.

**Table A5:** Muscle elasticity during the locomotion training protocol.

Variables	1 Session	5 Session	10 Session	15 Session	20 Session	η^2^	*p*-Value
**Sitting (LogD)**							
Rectus femoris	0.93 (0.05)	0.89 (0.06)	0.89 (0.08)	0.87 (0.06)	0.86 (0.14)	0.101	0.202
Tibialis anterior	0.68 (0.08)	0.70 (0.05)	0.70 (0.09)	0.67 (0.08)	0.63 (0.12)	0.154	0.082
Biceps femoris	1.02 (0.15)	1.06 (0.16)	1.05 (0.11)	1.06 (0.13)	1.02 (0.23)	0.037	0.532
Gastrocnemius	1.13 (0.18)	1.13 (0.17)	1.14 (0.11)	1.11 (0.17)	1.09 (0.37)	0.019	0.671
Deltoid	1.04 (0.08)	1.04 (0.12)	1.09 (0.12)	1.04 (0.10)	1.04 (0.24)	0.040	0.496
Upper trapezius	0.91 (0.11)	0.92 (0.12)	0.91 (0.12)	0.87 (0.10)	0.87 (0.20)	0.062	0.374
Middle trapezius	0.90 (0.08)	0.95 (0.07)	0.91 (0.08)	0.92 (0.10)	0.93 (0.16)	0.080	0.285
**Standing (LogD)**							
Rectus femoris	0.96 (0.07)	0.94 (0.10)	0.94 (0.12)	0.91 (0.12)	0.88 (0.18)	0.125	0.139
Tibialis anterior	0.63 (0.12)	0.65 (0.10)	0.67 (0.12)	0.65 (0.12)	0.62 (0.16)	0.082	0.279
Biceps femoris	1.13 (0.15)	1.17 (0.14)	1.15 (0.18)	1.13 (0.19)	1.06 (0.23)	0.115	0.154
Gastrocnemius	1.00 (0.18)	1.01 (0.17)	1.07 (0.15)	1.06 (0.15)	0.99 (0.28)	0.101	0.203
Deltoid	1.07 (0.09)	1.02 (0.12)	1.04 (0.14)	1.01 (0.13) *^‡‡^*	0.96 (0.18)	0.181	0.038
Upper trapezius	0.99 (0.17)	1.00 (0.17)	0.94 (0.17) *^†^*	0.92 (0.19) *^‡^*	0.84 (0.19)	0.261	0.013
Middle trapezius	1.03 (0.23)	1.01 (0.11)	1.01 (0.17)	0.97 (0.14)	0.92 (0.25)	0.103	0.189
**Wide-standing (LogD)**							
Rectus femoris	1.06 (0.15)	1.19 (0.14)	1.15 (0.14)	1.13 (0.15)	1.07 (0.25)	0.173	0.067
Tibialis anterior	0.55 (0.18)	0.59 (0.15)	0.60 (0.14)	0.50 (0.09) *^‡^*	0.46 (0.05)	0.233	0.036
Biceps femoris	1.18 (0.14)	1.20 (0.15)	1.20 (0.12)	1.19 (0.11)	1.17 (0.30)	0.007	0.882
Gastrocnemius	0.76 (0.11)	0.81 (0.05)	0.83 (0.04)	0.83 (0.05)	0.83 (0.15)	0.113	0.158
Deltoid	0.99 (0.10)	0.94 (0.09)	0.91 (0.08)	0.88 (0.08) *^‡^*	0.85 (0.20)	0.236	0.026
Upper trapezius	0.99 (0.10)	0.95 (0.09)	0.89 (0.07) *^†^*	0.87 (0.06) *^‡^*	0.87 (0.21)	0.200	0.038
Middle trapezius	1.23 (0.16)	1.26 (0.18)	1.06 (0.15) *^†††^*	1.04 (0.15)	0.99 (0.22)	0.528	<0.001

Data presented as mean (standard deviation); η^2^, partial eta square; *^†^* *p* < 0.05, *^†††^* *p* < 0.001, vs. 5-session; *^‡^ p* < 0.05, *^‡‡^ p* < 0.01 vs. 10-session.
